# Determination of T Cell Responses in Thai Systemic Sclerosis Patients

**DOI:** 10.1155/2022/5072154

**Published:** 2022-03-07

**Authors:** Oranit Likhit, Worawit Louthrenoo, Sa-nga Pattanakitsakul, Aroonroong Suttitheptumrong, Supot Hannongbua, Thanyada Rungrotmongkol, Hiroshi Noguchi, Fujio Takeuchi, Kobporn Boonnak

**Affiliations:** ^1^Department of Microbiology and Immunology, Faculty of Tropical Medicine, Mahidol University, Bangkok 10400, Thailand; ^2^Division of Rheumatology, Department of Internal Medicine, Faculty of Medicine, Chiang Mai University, 50200, Thailand; ^3^Division of Molecular Medicine, Research Department, Faculty of Medicine Siriraj Hospital, Mahidol University, Bangkok 10700, Thailand; ^4^The Center of Excellence in Computational Chemistry, Department of Chemistry, Faculty of Science, Chulalongkorn University, Bangkok 10330, Thailand; ^5^Biocatalyst and Environmental Biotechnology Research Unit, Department of Biochemistry, Faculty of Science, Chulalongkorn University, Bangkok 10330, Thailand; ^6^Program in Bioinformatics and Computational Biology, Graduate School, Chulalongkorn University, Bangkok 10330, Thailand; ^7^School of Pharmacy, Nihon Pharmaceutical University, Saitama 361-0806, Japan; ^8^School of Pharmaceutical Science, University of Shizuoka, Shizuoka 422-8526, Japan; ^9^Department of Immunology, Faculty of Medicine Siriraj Hospital, Mahidol University, Bangkok 10700, Thailand

## Abstract

**Objectives:**

This study is aimed at determining the role of T cells by assessing the numbers of IFN-*γ*- and IL-2-secreting T cells following stimulation with peptides derived from DNA topoisomerase-I protein in Thai SSc patients.

**Methods:**

Fifty Thai SSc patients and 50 healthy controls (HC) joined this study. IFN-*γ* and IL-2 levels upon stimulation of T cells with 6 peptides derived from DNA topoisomerase-I protein were determined. Anti-nuclear antibodies (ANA) and anti-Scl-70 antibodies were determined by using the ELISA method.

**Results:**

In SSc patients, we detected a significantly higher number of IFN-*γ*- and IL-2-secreting CD8^+^ T cells than IFN-*γ*- and IL-2-secreting CD4^+^ T cells after stimulation with pooled peptides derived from DNA topoisomerase-I protein. A similar percentage of CD4^+^IL-2^+^, CD4^+^IFN-*γ*^+^, and CD8^+^IL-2^+^ were detected following stimulation with DNA topoisomerase-I protein -in SSc patients with anti-Scl-70 antibody (SSc/anti-Scl-70^+^) and those without. In contrast, the amount of CD8^+^IFN-*γ*^+^ cells was significantly higher in SSc/anti-Scl-70^+^ than those without. Stimulation with individual peptides showed that CSLRVEHINLHPELD (sPep3; 15 amino acids; position 505-519 of DNA topoisomerase-I protein) was the optimal epitope that induced T cells secreting the highest levels of IFN-*γ* and IL-2. A higher percentage of IFN-*γ*^+^CD4^+^ T cells was detected in SSc/anti-Scl-70^+^ than those without the following stimulation with peptides 2 (amino acid position 475-486 [RAVALYFIDKLA] of protein DNA topoisomerase).

**Conclusion:**

The results from this study emphasize the critical role of DNA topoisomerase-I peptides on the activation of T cells in SSc patients. The findings provide a better understanding of SSc's immunopathogenesis and may lead to the development of diagnostic tools and specific treatments for SSc in the future.

## 1. Introduction

Systemic sclerosis (SSc), or scleroderma, is a chronic autoimmune inflammatory disease characterized by extensive tissue fibrosis and various vascular complications. The etiology of SSc remains unknown, but genetics, environmental factors, and immunological abnormalities have been found to contribute to the development of the disease. Among these factors, the immunological factors have been intensively studied. For instance, the presence of SSc-specific autoantibodies is commonly associated with the pathogenesis of SSc [1, 2]. Among these autoantibodies, antitopoisomerase has been reported to precede the development of overt clinical disease SSc patients [3, 4]. There are 2 types of SScs: limited cutaneous SSc (lcSSc) and diffuse cutaneous SSc (dcSSc) [5]. lcSSc patients usually have anti-centromere antibodies (ACA) and often present with pulmonary arterial hypertension, whereas dcSSc patients usually present with extensive skin involvement, cardiac involvement, and pulmonary fibrosis often associated with anti-topoisomerase-I autoantibodies (anti-Scl-70) [6]. Despite these robust associations, the level of serum anti-Scl-70 is not useful to monitor disease progression in SSc patients. Moreover, several studies also suggest that T cells play a vital role as a contribution factor for the development of SSc. For instance, the impairment of CD4^+^ T cell and regulatory T cell (Treg) function has been reported in SSc patients [7, 8]. High levels of CD8^+^ T cells were reported in a SSc patient's blood [9] and bronchoalveolar lavage fluid from SSc patients with lung fibrosis, suggesting the CD8^+^ T cells may play a critical role in the pathogenesis of SSc disease [10]. However, the explicit role of the CD8^+^ T cells on immunopathogenesis of SSc has not been intensively studied. Importantly, anti-DNA topoisomerase antibodies (anti-Scl-70) exhibit a strong association with human leukocyte antigen (HLA) alleles across different ethnic groups, suggesting the involvement of T cells in the autoantibody development [11, 12]. In this context, the identification of immunodominant T cell epitopes of the DNA topoisomerase-I protein is of particular relevance. The presence of T cells specific to DNA topoisomerase-I proteins has been investigated in SSc patients in several countries such as America, Europe, Korea, and Japan [13, 14]. There is limited data on T cell responses in SSc patients in Southeast Asia, including Thailand. Here, we studied T cell activation in Thai SSc patients by measuring the IFN-*γ*- and IL-2-secreting CD4^+^ and CD8^+^ T cells following stimulation with peptides derived from DNA topoisomerase-I protein [14]; the protein that has been reported to be related to anti-Scl-70 autoantibodies.

## 2. Materials and Methods

### 2.1. Patients and Healthy Volunteers

Fifty SSc patients who were followed up at the Division of Rheumatology, Department of Internal Medicine, Faculty of Medicine, Chiang Mai University, were included in the study. All SSc patients were diagnosed according to the 1980 American College of Rheumatology classification criteria for the diagnosis of systemic sclerosis [15]. The severity of skin involvement was determined by the Modified Rodnan Skin Score (MRSS) [16]. The fifty healthy volunteers used as control were medical personnel who did not have any evidence of autoimmune disease.

Approximately 20 mL of whole blood sample was collected in EDTA blood collection tubes from the SSc patients and healthy volunteers. The blood samples were transferred from Chiang Mai University to the Department of Microbiology and Immunology, Faculty of Tropical Medicine, Mahidol University, within 24 hours while being stored at 4-8°C.

### 2.2. PBMCs Isolation from Whole Blood Samples

PBMCs were isolated as previously described [17]. Briefly, whole blood samples were diluted 1 : 1 in PBS or RPMI 1640 medium (Life Technologies, Grand Island, NY) and layered over Ficoll-hypaque (Sigma-Aldrich, St. Louis, MO). The PBMC were washed repeatedly with PBS at lower *g* forces to remove platelets. The PBMC were then frozen in fetal bovine serum (FBS) (Life Technologies) supplemented with 10% dimethyl sulfoxide (Sigma-Aldrich). They were frozen at -80°C overnight and then transferred to the vapor phase of liquid nitrogen the next day. Before use, the cells were rapidly thawed at 37°C in complete medium (RPMI supplemented with 10% FBS, 2 mM-glutamine and 1X anti-anti (Life Technologies)). The cells were gently pelleted and resuspended with complete media for further use.

### 2.3. Candidate Peptides Derived from DNA Topoisomerase-I Protein

Six peptides were designed based on potential T cell epitopes of protein DNA topoisomerase-I (Scl 70) [12]. All 6 synthetic peptides were purchased from GenScript Japan Co. (Tokyo, Japan). The brief description and amino acid position of these six peptides are SPep1: NCSKDAKVPSPP (385-396), SPep2: RAVALYFIDKLA (475-486), SPep3: CSLRVEHINLHPELD (505-519), SPep4: KVVESKKKAVQRLEE (682-696), SPep5: PIEKIYNKTQREKFA (739-753), and SPep7: KFAWAIDMADED (751-762). These peptides have successfully stimulated T cells isolated from North American Caucasians, North American Blacks, and Japanese SSc patients in a previous study [12]. Therefore, we selected those peptides for testing in Thai SSc patients. A peptide Npep1 (LKRRIMPEDIIINCS) has been used as a control peptide. This peptide did not stimulate T cell responses in SSc patients and healthy volunteers similar to media control. Therefore, we used media control as a negative control for all experiments (Supplement Figure 1).

### 2.4. Antigen Stimulation and Intracellular Cytokine Staining (ICS)

Antigen stimulation and ICS were performed as previously described with some modification [18, 19]. 1x10^6^ PBMCs were stimulated with complete media containing 50 *μ*g/ml PMA and 250 ng/ml Ionomycin or 10 *μ*g/ml pooled peptides or 10 *μ*g/ml of individual peptides for 24 hours at 37°C in an incubator containing 5% CO_2_ and 5% humidity. During the last 16 hours of culture, 2 *μ*l of Golgistop™ (BD Biosciences, San Diego, CA) containing monensin (Biolegend, San Diego, CA) was added to block cytokine secretion. Cells were surface stained with anti-CD3-APC, anti- CD4-FITC, and anti-CD8 PerCP (BD Biosciences) for 30 minutes at 4°C. Following permeabilization, these cells were then stained with anti-human IFN-*γ* and IL-2 (BD Biosciences) for 30 minutes at 4°C. Cells were finally resuspended with 200 *μ*l of 2% paraformaldehyde (Sigma-Aldrich) and analyzed by Facscalibur (BD Biosciences). Data was analyzed using the CellQuest Pro software (BD Biosciences) and FlowJo 10.8.1 software (TreeStar, Ashland OR, USA). Gating is shown in supplementary Figure 1.

### 2.5. Ethical Statements

This study was approved by the ethical committee of the Faculty of Tropical Medicine, Mahidol University, Thailand (approval number: MUTM 2018-042-01); the ethical committee of Faculty of Medicine, Chiang Mai University, Thailand (approval number: MED-2559-04370/4370); and the ethical committee of Tokyo Seiei College, Japan (approval number: 20160004 and 20190006). All participants provided their written informed consent prior to entering the study.

### 2.6. Statistical Analysis

The normality of distribution of cytokine secretion levels were determined using Student's paired *t*-test. Summary statistic is expressed as median values. Data was analyzed using the GraphPad Prism software version 9.0 (GraphPad Software, Sam Diego, CA). Continuous variables were presented as mean ± standard deviation (SD) or median (min-max), while categorical variables were presented as frequency and percentage. To determine the difference between two independent samples of continuous variables, the Student *t*-test was used for variables with normal distribution and the Wilcoxon rank sum test for nonnormal distribution. A *p* value of less than 0.05 was considered statistically significant.

## 3. Results

### 3.1. Participant Demographic Data

There were 50 SSc patients (10 male and 40 female) who had a mean ± SD age of 50.4 ± 11.1 years, with the MRSS score ranging from 2 to 31 (median 7). There were 28 patients (56%) with dcSSc and 22 patients (44%) with lcSSc. Antinuclear antibody, anti-centromere antibody, and anti-topoisomerase antibody (anti-Scl70) presented in 50 (100%), 8 (16%), and 34 (68%) patients, respectively. The clinical manifestation of SSc was Raynaud's phenomenon in all (100%), lung involvement in 42 (84%), digital pitting scars in 27 (57%), dysphagia in 28 (56%), arthritis in 24 (48%), sclerodactyly in 23 (46%), telangiectasia in 22 (44%), and myositis in 3 (6%) patients. Family of SSc was documented in 3 patients (6%). For the control volunteers, 50 healthy volunteers (38 female and 12 male) who had a mean ± SD age of 58.9 ± 9.8 years were enrolled in the study. Among these healthy volunteers, antinuclear antibodies (ANA) were detected in 5 (10%) individuals.

### 3.2. Increasing Number of IL-2- and IFN-*γ*-Secreting T Cells following Stimulation with Pooled Peptides Derived from DNA Topoisomerase-I Protein in SSc Patients

**Figure 1:**
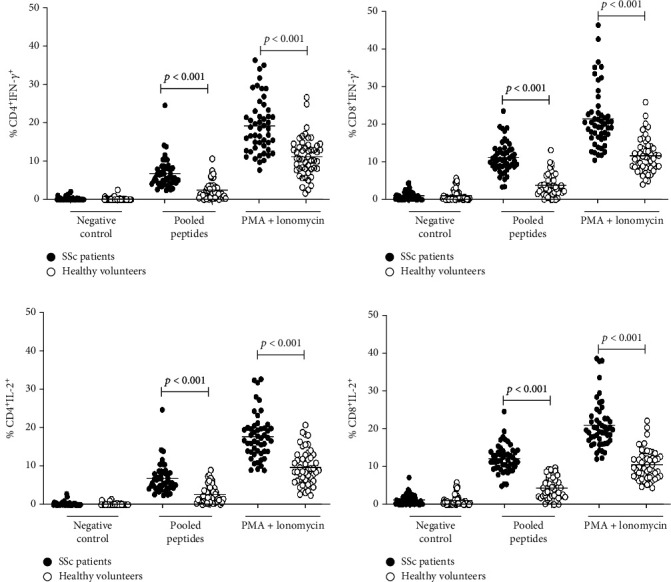
Percentage of cytokines (IFN-*γ* and IL-2) producing CD4^+^ (a, c) and CD8^+^ (b, d) T cells following stimulation with media (negative control), pooled peptides derived from DNA topoisomerase-I protein, and PMA ionomycin (positive control) in SSc patients (*n* = 50) and healthy volunteers (*n* = 50) (Student's *t*-test, *p* value < 0.001).

To determine T cell responses, the PBMCs from SSc patients were stimulated with 10 *μ*g/ml pooled peptides derived from topoisomerase protein (Scl-70 protein) for 24 hours. The combination of 250 ng/ml ionomycin and 50 *μ*g/ml PMA was used as a positive control stimulus in all experiments. We tested the T cell responses to other nonspecific peptide and found a similar degree of cytokine production as stimulated with media control (Supplementary Figure 2). Therefore, we used media control as a negative control for the following experiments. Following stimulation with the pooled peptides, we found increased numbers of IL-2 and IFN-*γ* secreting CD4^+^ and CD8^+^ T cells in the PBMCs isolated from SSc patients but not in healthy volunteers. Moreover, both CD4^+^ and CD8^+^ T cells from SSc patients responded to the PMA/ionomycin by secreting IL-2 and IFN-*γ* better than those detected in healthy volunteers (Figure 1).

**Figure 2:**
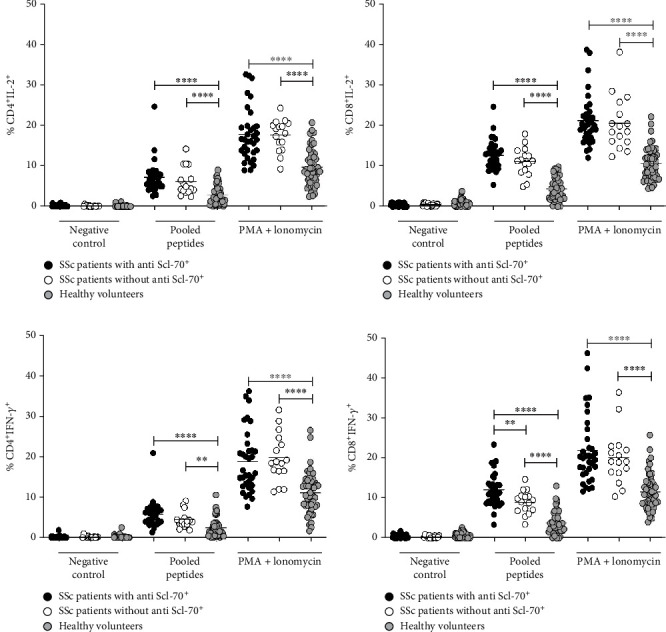
Percentage of cytokine (IFN-*γ* and IL-2) producing CD4^+^ (a, c) and CD8^+^ (b, d) T cells following stimulation with media (negative control), pooled peptides derived from topoisomerase protein, and PMA ionomycin (positive control) in SSc patients with positive anti-Scl70 antibodies (*n* = 34) and SSc patients with negative anti-Scl70 antibodies (*n* = 16) (Student's *t*-test, ∗∗∗∗*p* value < 0.001, ∗∗*p* value < 0.05).

We hypothesize that the level of anti-Scl-70 detected in SSc patient's serum is influenced by Scl-70 protein-specific T cells. Therefore, we determined whether the presence of anti-Scl-70 antibody is associated with the degree of cytokine production by T cells by stimulation with either PMA/ionomycin or pooled peptides derived from DNA topoisomerase-I protein. The results showed that a similar percentage of CD4^+^IL-2^+^, CD4^+^IFN-*γ*^+^, and CD8^+^IL-2^+^ T cells was detected in SSc patients with and without anti-Scl-70 antibody following stimulation with peptides derived from Scl-70 protein (Figures 2(a)–2(c)). Interestingly, the amount of CD8^+^IFN-*γ*^+^ T cells was significantly higher in SSc patients with anti-Scl-70 antibody (Figure 2(d)) following stimulation with pooled peptides but not with PMA ionomycin. This result suggests that the presence of Scl-70 antibodies may be related to the production of IFN-*γ* by T cells and vice versa.

**Figure 3:**
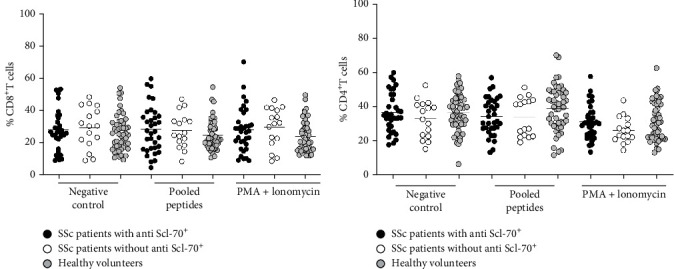
The number of CD4^+^ (a) and CD8^+^ (b) T cells in SSc patients (*n* = 50) and healthy volunteers (*n* = 50).

To exclude the possibility that the numbers of T cells were different among SSc patients and healthy volunteers; which may contribute to the difference in the percentage of the cytokine-producing T cells. The enumeration of CD4^+^ and CD8^+^ T cells was performed by flow cytometry in both SSc patients and healthy volunteers in all conditions tested in this study. Both groups showed a similar percentage of CD4^+^ and CD8^+^ T cells (approximately 30%, mean ± SD = 29.55 ± 9.29) (Figure 3), regardless of the experiment condition. This data indicated that the percentage of IFN-*γ*^+^ and IL-2^+^ secreted from T cells observed in subsequent experiments was not a result of differences in the percentage of CD4^+^ and CD8^+^ T cells among SSc patients with and without anti-Scl-70 antibody and healthy volunteers.

### 3.3. Similar T Cell Responses Were Observed in dcSSc and lcSSc

**Figure 4:**
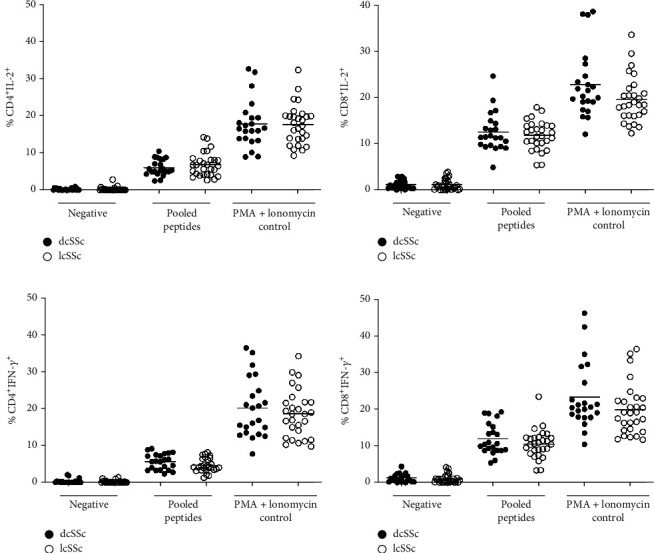
Percentage of cytokines (IFN-*γ* and IL-2) producing CD4^+^ (a, c) and CD8^+^ (b, d) T cells following stimulation with media (negative control), pooled peptides derived from DNA topoisomerase-I protein, and PMA ionomycin (positive control) in dcSSc (*n* = 22) and lcSSc (*n* = 28) patients.

In order to explore the difference in T cell responses in patients with dcSSc and lcSSC, the percentage of cytokine secreting CD4^+^ and CD8^+^ T cells in these two groups were compared. The percentage of CD4^+^IFN-*γ*^+^-, CD4^+^IL-2^+^-, CD8^+^IFN-*γ*^+^-, and CD8^+^IL-2^+^-secreting cells was similar between dcSSc and lcSSc patients following stimulation with positive control stimulus and pooled peptides from DNA topoisomerase-I protein. No significant difference in T cell responses among these two groups was observed (Figure 4). Therefore, the level of IFN-*γ* and IL-2 production by T cells is not directly associated with the type of SSc disease.

### 3.4. High Levels of IFN-*γ* and IL-2 Expression Were Observed from the CD8^+^ T Cells of SSC Patient with Anti-SCl-70 Antibody

**Figure 5:**
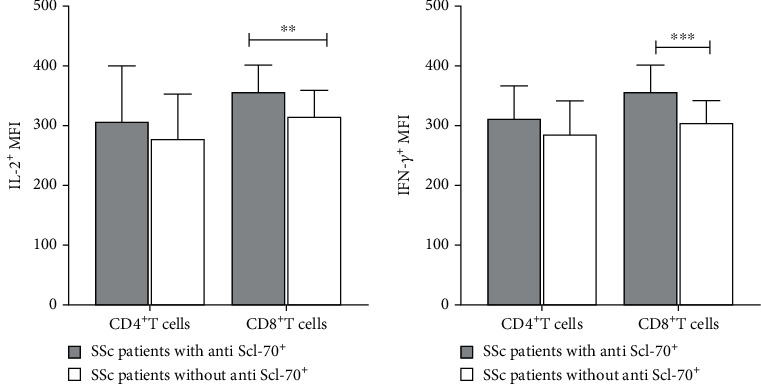
Interleukin-2 (a) and interferon gamma (b) expression of CD4^+^ and CD8^+^ T cells from SSc patients with and without anti-Scl-70 antibody following stimulation with pooled peptides derived from DNA topoisomerase-I protein. Data was expressed as mean fluorescence intensity (MFI) + SEM of positive cells (Student's *t*-test, ∗∗*p* < 0.01 and ∗∗∗*p* < 0.001 (*n* = 34; SSc patients with anti-Scl-70 antibody, *n* = 16; SSc patients without anti-Scl-70 antibody).

Expression of IFN-*γ* and IL-2 by CD4^+^ and CD8^+^ T cells following stimulation with pooled peptides was determined. Mean fluorescence intensity (MFI) was used to quantify CD4^+^ and CD8^+^ T cells which stained positive for IFN-*γ* and IL-2. The MFI of IFN-*γ* and IL-2 was similar in CD4^+^ T cells from anti-Scl-70 positive and negative SSc patients (IFN-*γ*; 312.4 ± 54.29 vs 285.60±56.27 and IL-2; 314.70±76.34 vs 285.60±56.27). In contrast, the MFI of IFN-*γ* and IL-2 was significantly higher in CD8^+^ T cells of SSc patients with anti-Scl-70 antibody than that in patients without anti-Scl-70 antibody (IFN-*γ*; 357.00 ± 43.6 vs 303.00 ± 38.70; *p* < 0.001 and IL-2; 356.60 ± 44.71 vs 314.30 ± 44.73; *p* < 0.01) (Figure 5).

### 3.5. IFN-*γ* and IL-2 Secreting CD4^+^ and CD8^+^ T Cells following Stimulation with Individual Peptides Derived from DNA Topoisomerase-I Protein

**Figure 6:**
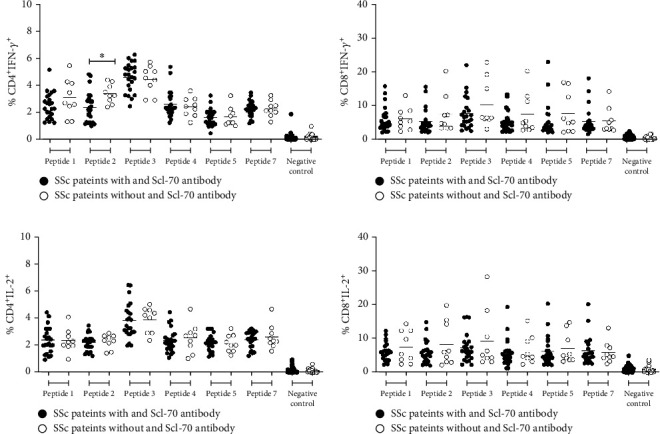
Percentage of cytokine (IFN-*γ* and IL-2) producing CD4^+^ (a, c) and CD8^+^ (b, d) T cells following stimulation with individual peptides in SSc patients (*n* = 32), (Student's *t*-test, ∗*p* < 0.05).

To further identify the specific epitopes among these six peptides derived from DNA topoisomerase-I protein, the available PBMCs isolated from 23 SSc patients with anti-Scl-70 antibody and 9 SSc patients without anti-Scl-70 antibody were stimulated with individual peptides at the concentration of 10 *μ*g/ml for 24 hours. The percentage of IFN-*γ*- and IL-2-secreting T cells was determined by flow cytometry. The results revealed higher percentage of IFN-*γ* and IL-2 secreting CD8^+^ T cells than that observed in CD4^+^ T cells following stimulation with individual peptides (Figures 6(a)–6(d)). Interestingly, higher number of IFN-*γ*^+^ CD4^+^ T cells isolated from SSc patients who were anti-Scl-70 antibody negative was observed than that in CD4^+^ T cells from SSc patients with anti-Scl-70 antibody following stimulation with peptides 2, which corresponds to amino acid position 475-486 (RAVALYFIDKLA) of protein DNA topoisomerase-I (Figure 6(a)). We did not observe a significant difference in the percentage of IL-2 producing CD4^+^ T cell between these two groups. Although high numbers of IL-2^+^CD8^+^ and IFN-*γ*^+^CD8^+^ T cells were observed in SSc patients, there was no significant difference in the levels of these cells in SSc patients with or without anti-Scl-70 antibody.

## 4. Discussion

Although the role of T cells in the development of systemic sclerosis has long been recognized, the mechanisms responsible for the initiation of T cells responses in the pathogenesis of SSc remain insufficiently understood. Here, we demonstrated the effect of DNA topoisomerase-I protein on T cell activation in Thai SSc patients. The DNA topoisomerase-I protein was chosen because it has been shown to affect immune activation in SSc patients [12]. Since T cells are necessary for the production of anti-topoisomerase-I antibodies in SSc patients [12], we sought to determine the T cell responses following stimulation with peptides derived from DNA topoisomerase-I proteins in Thai SSc patients with and without anti-Scl-70 antibodies. In this study, we focus on IFN-*γ* and IL-2 secreting CD4^+^ and CD8^+^ T cells because high levels of IFN-*γ* have been observed in SSc patient's plasma samples [20, 21]. Additionally, IL-2 is involved in the proliferation of T cells and affects several immunological pathways [22]. However, the controversy of IL-2 levels in SSc patient serums has long been recognized [5, 23–25]. This might be due to different disease status in the same patient and different population study groups. Moreover, genetic alteration of IL-2 gene has been proposed as a marker for the limited phenotype of SSc [26].

Although the association of anti-Scl-70 antibody and the presence of topoisomerase-I (Scl-70) specific CD4^+^ T cells in SSc patients has been reported [27], we found that following stimulation by peptides derived from DNA topoisomerase protein, the numbers of IFN-*γ* and IL-2 secreting CD4^+^ T cells was comparable in SSc patients with and without anti-Scl-70 antibodies. This might be due to a different antigen used to activate PBMCs and/or use of different study populations. In regards to the activation of CD8^+^ T cells, a higher percentage of IFN-*γ* secreting CD8^+^ T cells was observed in SSc patients with anti-Scl-70 antibodies following stimulation with peptides derived from DNA topoisomerase-I. On the other hand, similar levels of IL-2 secreting CD8^+^ T cells were observed in both groups. However, conflicting results have been reported so far as some studies found that PHA stimulated PBMCs from SSc patients produced less IFN-*γ* than the control group [24, 28] whereas a study from Italy showed increased levels of IFN-*γ* mRNA in the peripheral blood of SSc patients following PMA stimulation when compared to healthy controls [29]. In agreement with other studies investigating the cytokine production following stimulation with PMA and ionomycin [28–30], we also found that T cells isolated from SSc patients secreted IFN-*γ* and IL-2 better than the T cells isolated from healthy volunteers. Increased levels of interferon (IFN) regulated genes such as *Siglec 1* and *MX1* have been reported in monocytes isolated from lcSSc patients, especially lcSSc associated with pulmonary arterial hypertension [31]. However, we observed a similar percentage of IFN-*γ*-secreting T cells between lcSSc and dcSSc patients following stimulation with DNA topoisomerase-I peptide. The discrepancy may be caused by different cell types and antigens used in the assay. The contribution of IFN-*γ* as biomarkers for SSc to distinguish different types of SSc patients warrants further research. Our results indicated that IFN-*γ* and IL-2 were predominantly secreted by CD8^+^ T cells in SSc patients and healthy volunteers following stimulation with DNA topoisomerase-I peptides or control antigen. We therefore speculate that these antigens might be potent activators for CD8^+^ T cells, but the activation mechanism needs to be further studied.

We found that peptide 3 (Spep3, CSLRVEHINLHPELD; length 15 amino acids; amino-acid position 505-519 of DNA topoisomerase protein), followed by peptides 2 and 4, efficiently stimulated CD4^+^ and CD8^+^ T cells of SSc patients. Interestingly, stimulation with peptide 2, which corresponds to amino acid position 475-486 (RAVALYFIDKLA) of DNA topoisomerase-I protein, induced a higher percentage of IFN-*γ* secreting CD4^+^ T cells in SSc patients without anti-Scl-70 antibody than that observed in patients with anti-Scl-70 antibodies. This effect has not been detected by stimulation with other individual peptides, suggesting that this peptide may contribute to T cell responses in the SSc patients. However, further studies need to be conducted to understand the biological function of peptide 2. Majority of the peptides used in our study were between 12-15 amino acids long. Theoretically, this length is suitable for MHC class II processing and activation of CD4^+^ T cells. Peptides 2, 3, and 4 contain 12-15 amino acids which should be efficiently processed and presented to T cells in the context of MHC-II. However, we found that CD8^+^ T cells from SSc patients reacted to these peptides by secreting cytokines more efficiently than CD4^+^ T cells. This phenomenon has been demonstrated in a study on mice, which showed that long peptides could be presented by the MHC class I molecule [32]. Chen et al. found a high affinity of naturally processed peptides in different amino acids; 9-mer, 10-mer, and 12 mer peptides with human MHC class I molecule (HLA-A2.1) [33]. This data suggests that different naturally occurring longer peptides can bind in different conformations to MHC class I molecules. Probst-Kepper et al. previously showed that the pockets A and F of HLA-B∗35 : 01 groove in humans have a high affinity and bind with long peptides over 14 amino acids [34]. Moreover, a study from Australia demonstrated that peptides of >10 residues could be recognized by MHC class I molecule. They determined the T cell cytotoxicity in the context of HLA-B35 upon stimulation with 11 or 12 amino acid residues peptides derived from the Epstein-Barr virus (EBV) [35]. All these evidences support our finding that CD8^+^ T cells can be triggered by longer peptides and possibly via the MHC class I molecule. Other evidence that supports this hypothesis is that further processing of the 15 mers peptides occurs in the *in vitro* culture leading to smaller peptides that can be accommodated in the MHC class I groove in the normal fashion [36, 37].

Our study also has some limitations. We only focused on Th1 cytokines (IFN-*γ* and IL-2) whereas other investigators suggested the association of Th2 responses and SSc pathogenesis. Indeed, the Th2 cytokine response, with production of IL-4, IL-10, and TGF-*β*, leads to tissue fibrosis [38, 39]. The small sample size might be an issue for statistical analysis. Future studies with a larger sample size are required to confirm our findings. In addition, determination of both Th1 and Th2 cytokine functions in response to specific peptide stimulation in SSc patients would also be of interest.

## 5. Conclusion

In conclusion, we found increased levels of IFN-*γ* and IL-2 secreting CD4^+^ and CD8^+^ T cells upon stimulation with the DNA topoisomerase-I peptides. This cytokine production following stimulation with the peptides was restricted to SSc patients and not healthy volunteers. These results amplify the finding of T cell responses to DNA topoisomerase-I which is an important factor in the pathogenesis of SSc and may be the target of future therapeutic interventions.

## Data Availability

The datasets used and/or analyzed of this study are available from the corresponding author on reasonable request.

## Supplementary Materials

Supplementary 1 Supplementary Figure 1. Gating strategy. PBMCs from healthy donors (A and B) and SSc patients (C and D) and stimulated with pooled peptides derived from DNA topoisomerase-I protein then subjected to intracellular staining for CD4+IFN*γ*+, CD4+IL-2+, CD8+IFN*γ*+, and CD8+IL-2+ events.

Supplementary 2 Supplementary Figure 2. Percentage of cytokines (IFN-*γ* and IL-2) producing CD4^+^ (A and C) and CD8^+^ (B and D) T cells following stimulation with media (negative control) and nonspecific peptide.
