# Supplementary material for: Determination of T Cell Responses in Thai Systemic Sclerosis Patients
Source: J Immunol Res. 2022 Mar 7;2022:5072154. doi: 10.1155/2022/5072154 (PMC8924789; doi:10.1155/2022/5072154)
Supplement: Supplementary 1 — Supplementary Figure 1. Gating strategy. PBMCs from healthy donors (A and B) and SSc patients (C and D) and stimulated with pooled peptides derived from DNA topoisomerase-I protein then subjected to intracellular staining for CD4+IFNγ+, CD4+IL-2+, CD8+IFNγ+, and CD8+IL-2+ events. [file 5072154.f1.docx]

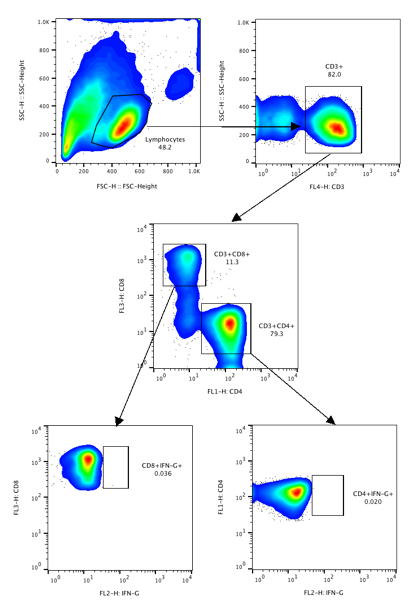

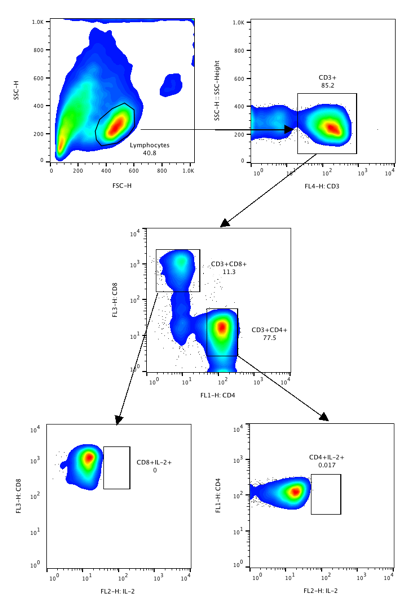


A...

D.

B.

CD4^+^IFNγ^+^

CD4^+^IL-2^+^

CD8^+^IL-2^+^

CD8^+^IFNγ^+^

C.


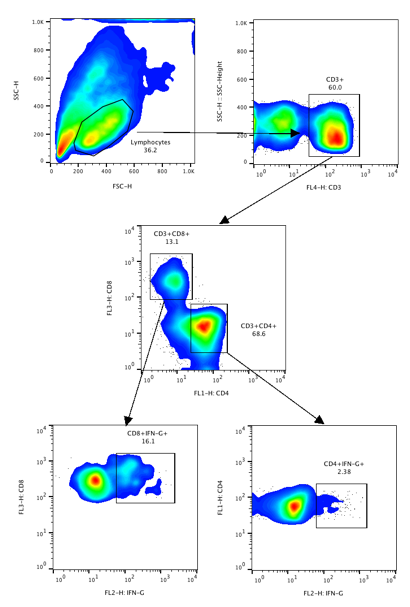

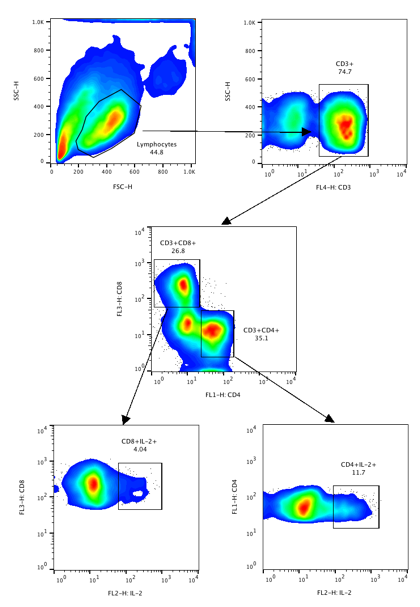


CD4^+^IFNγ^+^

CD8^+^IFNγ^+^

CD8^+^IL-2^+^

CD4^+^IL-2^+^

**Supplementary Figure 1.** Gating strategy. PBMCs from healthy donors (A and B) and SSc patients (C and D) and stimulated with pooled peptides derived from DNA topoisomerase I protein then subjected to intracellular staining for CD4^+^IFNγ^+^, CD4^+^IL-2^+^, CD8^+^IFNγ^+^, and CD8^+^IL-2^+^ events.
