# Supplementary material for: Determination of T Cell Responses in Thai Systemic Sclerosis Patients
Source: J Immunol Res. 2022 Mar 7;2022:5072154. doi: 10.1155/2022/5072154 (PMC8924789; doi:10.1155/2022/5072154)
Supplement: Supplementary 2 — Supplementary Figure 2. Percentage of cytokines (IFN-γ and IL-2) producing CD4+ (A and C) and CD8+ (B and D) T cells following stimulation with media (negative control) and nonspecific peptide. [file 5072154.f2.docx]

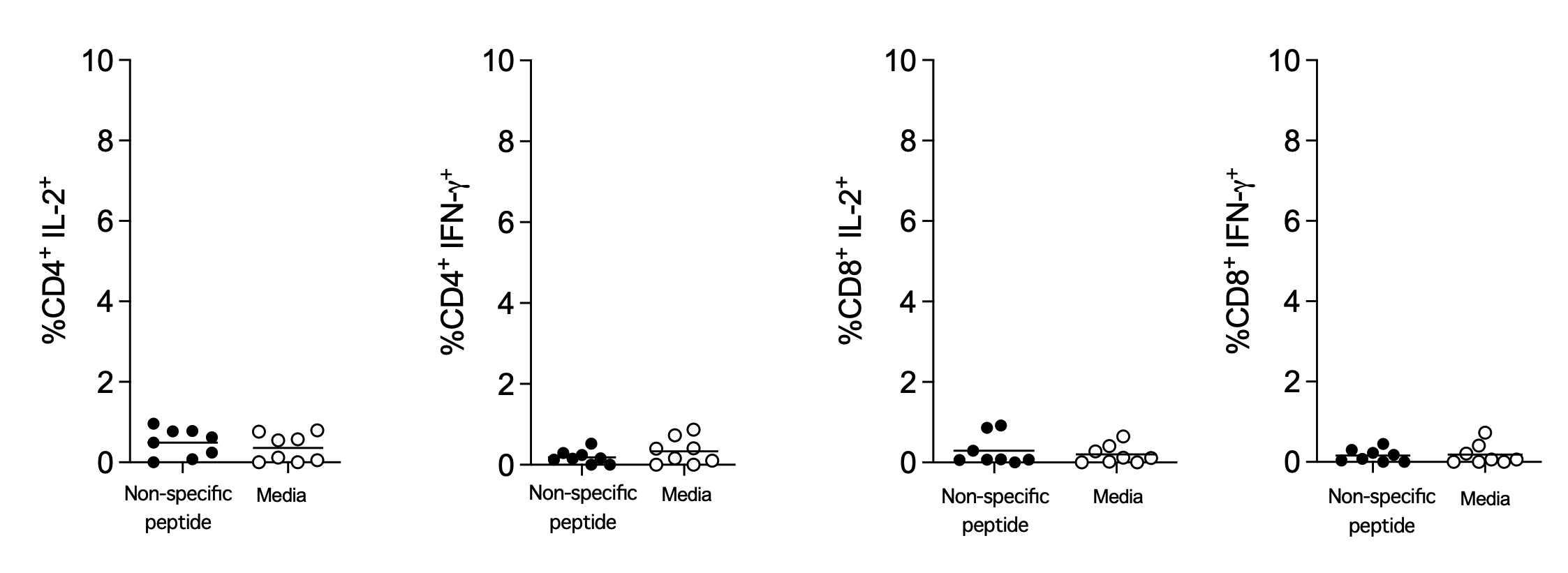


**Supplementary Figure 2.** Percentage of cytokines (IFN-γ and IL-2) producing CD4^+^ (A and C) and CD8^+^ (B and D) T cells following stimulation with media (negative control), and non-specific peptide.
